# Platform-Dependent Differences in Beam Characteristics and Low-Dose Exposure: A Comparative Study of Elekta™ Synergy and Varian TrueBeam™ Linear Accelerators Using SunSCAN™ 3D Phantom and Octavius^®^ 4D QA

**DOI:** 10.3390/jcm15041619

**Published:** 2026-02-20

**Authors:** Marian-Răzvan Bălan, Anda Elena Crișan, Eugen Osiac, Cristiana-Iulia Dumitrescu, Suzana Măceș, Mihai Popescu, Luana Corina Lascu, Maria Mihai, Sanda-Amelia Drăcea, Oana Ciobănescu, Mădălin-Cristian Moraru, Daniela Dumitrescu

**Affiliations:** 1 Doctoral School, University of Medicine and Pharmacy of Craiova, 200349 Craiova, Romania; balan.razvan796@gmail.com (M.-R.B.); oana.ciobanescu@yahoo.com (O.C.); mmc9292@gmail.com (M.-C.M.); 2 Department of Radiotherapy, University Emergency County Clinical Hospital Craiova, 200642 Craiova, Romania; mariamihai953@yahoo.com (M.M.); sanda.amelia.dracea@gmail.com (S.-A.D.); 3 SC SPAD Imaging International SRL Center, 200352 Craiova, Romania; macessuzana@yahoo.com (S.M.); mihai_popescu_rad@yahoo.com (M.P.); luanalascu67@gmail.com (L.C.L.); daniela.dumitrescu@gmail.com (D.D.); 4 Department of Oncology and Radiotherapy, University of Medicine and Pharmacy of Craiova, 200349 Craiova, Romania; 5 Department of Biophysics, University of Medicine and Pharmacy of Craiova, 200349 Craiova, Romania; 6 Department of Pharmacology, Faculty of Medicine, University of Medicine and Pharmacy of Craiova, 200349 Craiova, Romania; dumitrescu.cristiana@gmail.com; 7 Department of Radiology and Medical Imaging, University of Medicine and Pharmacy of Craiova, 200349 Craiova, Romania; 8 Department of Radiology and Medical Imaging, University Emergency County Clinical Hospital, 200642 Craiova, Romania

**Keywords:** VMAT, IMRT, Elekta Synergy, Varian TrueBeam, multileaf collimator, penumbra, low-dose bath, OAR dosimetry, SunSCAN™ 3D, Octavius^®^ 4D

## Abstract

**Background/Objectives:** Inter-platform variability in beam characteristics and low-dose exposure may arise from differences in linear accelerator head design, multileaf collimator geometry, and dose calculation algorithms. This study aimed to evaluate system-level dosimetric differences between two widely used linear accelerator platforms under clinically commissioned conditions. **Methods:** A comparative dosimetric analysis was performed between Elekta Synergy and Varian TrueBeam linear accelerators. Beam data were acquired using a SunSCAN™ 3D water phantom, and patient-specific quality assurance was conducted with the Octavius^®^ 4D system. Treatment plans were generated for left-sided breast, prostate, and head and neck cases using clinically commissioned treatment planning systems. Beam flatness, symmetry, penumbra width, low-dose exposure, conformity, homogeneity, and organ-at-risk dose metrics were evaluated. **Results:** Platform-dependent differences were observed in penumbra behavior and out-of-field dose, primarily attributable to intrinsic linac head design and collimation characteristics. These differences propagated into clinical plans, with greater variability observed for breast and head and neck cases, while prostate plans showed higher consistency between platforms. Algorithm-dependent trends were noted for conformity and homogeneity indices; however, all plans met institutional clinical acceptance criteria during quality assurance. Stricter gamma evaluation criteria revealed systematic but limited inter-platform deviations. **Conclusions:** Elekta Synergy and Varian TrueBeam demonstrated clinically acceptable dosimetric performance, with modest platform-dependent differences. While target coverage and overall plan quality were comparable, these variations were primarily observed in peripheral dose regions and may be relevant for platform-specific planning optimization and quality assurance. This supports the importance of comprehensive commissioning and QA procedures in both mixed- and single-platform clinical settings, particularly for highly modulated techniques.

## 1. Introduction

Radiotherapy remains a cornerstone of modern cancer management, with continuous technological advances aimed at improving dose conformity to the target volume while minimizing exposure to surrounding healthy tissues. The widespread implementation of intensity-modulated radiotherapy (IMRT) and volumetric modulated arc therapy (VMAT) has significantly increased treatment complexity, placing greater demands on beam modeling accuracy, treatment planning systems (TPS), and quality assurance (QA) procedures [1,2,3,4]. Early comparative studies of commercial TPS platforms demonstrated that differences in beam modeling algorithms and system-specific assumptions may lead to measurable dosimetric discrepancies, particularly in regions outside the high-dose target volume [1]. As treatment techniques have evolved toward highly modulated delivery, the accurate characterization of beam profiles, penumbra, and out-of-field dose has become increasingly important for both plan optimization and comprehensive QA frameworks [2].

### 1.1. Beam Modeling and Profile Characterization

Beam profile characteristics, including flatness and symmetry, represent fundamental indicators of linear accelerator performance and are routinely evaluated during commissioning and periodic QA [2]. Several experimental and multi-institutional studies have reported that modern linear accelerators can achieve highly stable and reproducible beam profiles when appropriate commissioning protocols are applied [5,6,7,8].

However, subtle differences in beam profiles may still arise from platform-dependent factors, including collimator geometry, source-size modeling, and algorithmic implementation within the TPS [9,10]. These differences, although often clinically acceptable, may become relevant in highly conformal treatment techniques where steep dose gradients are present.

### 1.2. Penumbra and Small-Field Considerations

The penumbra region, defined by the dose transition at the field edge, is particularly sensitive to collimation design, multileaf collimator (MLC) characteristics, and source-to-surface distance. Previous investigations have demonstrated that jaw-tracking techniques, MLC positioning accuracy, and field geometry significantly influence penumbra width and dose fall-off, particularly in small and modulated fields [5,11].

Monte Carlo–based studies further suggest that observed penumbral differences are predominantly driven by mechanical and geometric factors rather than inaccuracies in dose calculation algorithms [9,12]. Given the increasing use of small and irregular fields in VMAT, systematic evaluation of penumbra behavior remains a critical component of beam characterization.

### 1.3. Out-of-Field Dose and Low-Dose Exposure

Beyond the high-dose treatment region, out-of-field dose—often referred to as the low-dose bath—has gained increasing attention due to its potential long-term clinical implications. Peripheral dose arises from a combination of head scatter, collimator transmission, and leakage radiation, all of which are influenced by linac head design and shielding configuration [5,6].

Several clinical and dosimetric studies have highlighted the relevance of low-dose exposure to radiosensitive organs, particularly in breast cancer radiotherapy, where incidental cardiac and pulmonary irradiation has been associated with increased long-term morbidity [13,14,15]. As a result, a detailed assessment of out-of-field dose has become increasingly relevant in the comparative evaluation of treatment platforms.

### 1.4. Platform-Dependent Effects and Treatment Complexity

Modern radiotherapy delivery is inherently platform-dependent, with differences in MLC design, delivery mechanics, and TPS optimization strategies contributing to variations in plan complexity and dosimetric outcomes. Multi-institutional audits and knowledge-based planning studies have demonstrated that accelerator platform and planning system selection may influence modulation complexity, delivery accuracy, and QA performance [16,17].

End-to-end testing approaches further underscore the importance of comprehensive system-level evaluation, demonstrating that small systematic differences in beam modeling and delivery can propagate throughout the entire treatment chain [18]. These findings underscore the need for comparative, platform-aware dosimetric studies that integrate physical beam characterization with treatment planning evaluation.

### 1.5. Clinical Relevance and Organ-at-Risk Considerations

In addition to target dose conformity, organ-at-risk (OAR) sparing remains a central objective of contemporary radiotherapy. Even modest reductions in low-dose exposure to critical structures such as the heart, lungs, spinal cord, and contralateral breast may contribute to reduced late toxicity risk [13,14,15,19].

From a regional and clinical perspective, previous work by Anghel and collaborators has emphasized the role of advanced imaging, individualized dosimetric assessment, and careful treatment planning in minimizing unnecessary organ exposure, particularly in breast cancer radiotherapy [20,21,22]. These considerations further motivate the need for a detailed comparative evaluation of beam characteristics and dose distribution across different treatment platforms.

### 1.6. Aim of the Study

This study aimed to perform a comprehensive comparative dosimetric evaluation of Elekta Synergy and Varian TrueBeam linear accelerators. The analysis focused on beam profile characteristics, including flatness and symmetry, penumbra behavior, and out-of-field dose, complemented by treatment planning and organ-at-risk evaluation for representative clinical scenarios. By integrating physical beam measurements with test plan analysis, this work seeks to provide insight into platform-dependent dosimetric characteristics and their potential implications for treatment planning and quality assurance.

## 2. Materials and Methods

### 2.1. Irradiation Equipment and Treatment Planning Systems

This study employed two widely implemented linear accelerator (linac) platforms—Varian™ TrueBeam and Elekta™ Synergy—selected for their distinct head architectures, MLC designs, and dose-calculation workflows. The present investigation was designed as a system-level dosimetric comparison reflecting real-world clinical practice across different treatment platforms. Consequently, differences in linac hardware, treatment planning systems, and dose calculation algorithms were intentionally preserved rather than normalized. The aim of the study was not to isolate the individual contribution of hardware or algorithmic components, but to evaluate the combined dosimetric behavior encountered in routine clinical environments. Prior reports have demonstrated that these system-level differences can influence small-field dosimetry, penumbra sharpening, MLC transmission, and low-dose out-of-field behavior [1,6,11,16].

#### 2.1.1. Varian TrueBeam (6 MV, FF)

The Varian TrueBeam linac (Varian Medical Systems, Palo Alto, CA, USA) operated in 6 MV flattened (FF) mode using the jaws and Millennium 120 MLC (5 mm central, 10 mm peripheral leaves). Previous inter-platform studies have shown that the Millennium MLC introduces predictable variations in leaf-end transmission and tongue-and-groove behavior, particularly relevant for modulated deliveries [18,21]. The system allowed a maximum 40 × 40 cm^2^ field size.

Treatment planning was performed in Eclipse v17.1 using:

- AAA (Analytical Anisotropic Algorithm), known for robust performance in homogeneous geometries
- Acuros XB v13.5, an advanced deterministic solver validated in multiple independent studies for improved modeling of lateral transport and heterogeneous media [12,20].

#### 2.1.2. Elekta Synergy (6 MV, FF)

The Elekta Synergy linac (Elekta AB, Stockholm, Sweden) delivered 6 MV FF beams using the Agility MLC, characterized by 5 mm leaves, reduced transmission, and rapid leaf speeds—properties shown to impact both penumbra width and IMRT/VMAT modulation efficiency [9,14,23,24].

Treatment planning was performed in Monaco Planning System v6.22 using:

- Monte Carlo (MC) as the primary dose engine, the gold standard for complex geometries and small-field scenarios [23];
- Collapsed Cone (CC) for model validation and consistency assessment.

Both linacs used jaws and MLC, or MLC alone (Elekta’s case), with a maximum opening of 40 × 40 cm^2^.

### 2.2. Dosimetric System and Calibration Protocol

#### 2.2.1. Phantom and Detectors

**Figure 1:**
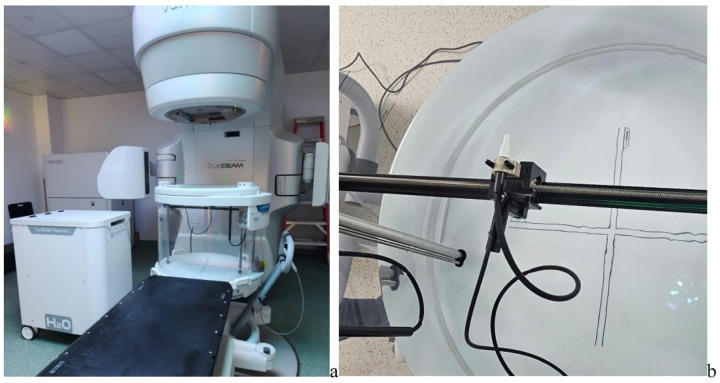
(**a**) SunScan™ 3D phantom setup; (**b**) water-filled phantom in Elekta site.

All measurements were acquired using the Sun Nuclear Corporation, 3275 Suntree Blvd, Melbourne, FL 32940, USA, systems SunSCAN™ 3D Water Phantom, which enables simultaneous inline, crossline, and depth scanning. Its high detector density has been previously used in commissioning and cross-platform comparison studies, as in Figure 1a,b [16,25,26].

The central axis (CAX) was defined as the geometric beam center (0 cm) for all scans. Lateral beam profiles were acquired in both inline and crossline directions.

Square field sizes of 3 × 3 cm^2^, 5 × 5 cm^2^, 10 × 10 cm^2^, and 30 × 30 cm^2^ were evaluated. For comparative analysis between platforms, particular emphasis was placed on the 10 × 10 cm^2^ field, which served as the reference field size for profile characterization, penumbra assessment, and out-of-field dose evaluation.

Additional profiles were acquired at dmax, 5 cm, and 30 cm depths to verify depth-dependent consistency; however, only the 10 cm depth data are presented, as these were deemed most representative for inter-platform comparison and clinical relevance.

Absolute calibration followed IAEA TRS-398 and AAPM TG-51 recommendations, performed with a waterproof Sun Nuclear Corporation, 3275 Suntree Blvd, Melbourne, FL 32940, USA, SNC™ Farmer ionization chamber (600 cc) at 10 cm depth [27,28].

#### 2.2.2. Scanning Detectors

Beam profiles and penumbra characterizations were collected using:

- Sun Nuclear Corporation, 3275 Suntree Blvd, Melbourne, FL 32940, USA, SNC™125c field and reference detectors;
- Sun Nuclear Corporation, 3275 Suntree Blvd, Melbourne, FL 32940, USA, Sun Nuclear Edge Detector™.

**Figure 2:**
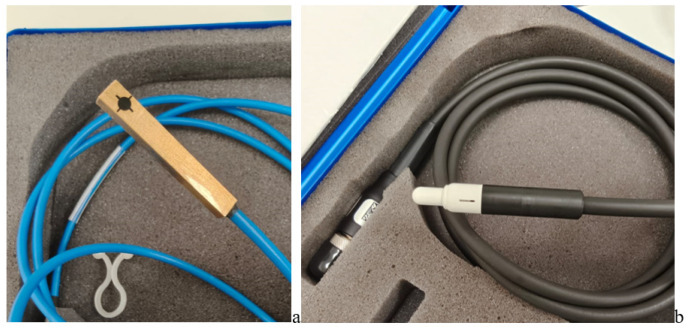
Detectors—(**a**) SNC™125c; (**b**) SNC Edge detector™.

These detectors, shown in (Figure 2a,b), are widely adopted for small-field profiling and high-gradient regions [11,18].

#### 2.2.3. Measurement Conditions

The two linear accelerator platforms are commissioned using different reference geometries. Varian TrueBeam measurements were performed at a source-to-surface distance (SSD) of 100 cm. In comparison, Elekta Synergy measurements were acquired at an SSD of 90 cm, consistent with manufacturer-specific commissioning protocols. Beam data acquisition was performed using an isocentric source-to-axis distance (SAD) geometry. Accordingly, all measurements were referenced to equivalent depths relative to the isocenter, ensuring dosimetric comparability between platforms despite differences in commissioning geometry.

While equivalent depths relative to the isocenter were maintained, the use of different source-to-surface distances may introduce geometric scaling effects that can influence projected field size, penumbra width, and lateral dose sampling near the field edge. This effect is particularly relevant for dose values sampled at ±5 cm from the central axis, which correspond closely to the geometric field edge. These potential geometric influences were therefore taken into account when interpreting lateral distance–based comparisons between platforms.

All scans were conducted at 10 cm depth, except percentage depth dose (PDD) acquisitions (continuous depth acquisition). Temperature, pressure, and electrometer corrections were applied in accordance with standard dosimetry protocols.

### 2.3. Comparative Physical Measurements

#### 2.3.1. Penumbra and Beam Edge Characteristics

Lateral profiles at 10 cm depth were collected for square fields:

- 3 × 3 cm^2^;
- 5 × 5 cm^2^;
- 10 × 10 cm^2^;
- 20 × 20 cm^2^;
- 30 × 30 cm^2^.

Penumbra width (PW) was defined as the lateral distance between 80% and 20% isodose points, following methodology similar to earlier cross-platform evaluations [2,29,30].

Penumbral characteristics were evaluated using lateral beam profiles acquired at a depth of 10 cm. Penumbral behavior was assessed for both inline and crossline directions, with particular attention to the ±5 cm region from the central axis for the 10 × 10 cm^2^ field, corresponding to the nominal geometric field edge. Left–right symmetry of the penumbra was also qualitatively evaluated to identify potential asymmetries in beam shaping.

$$D_{\pm 5}=\frac{D_{-5}+D_{+5}}{2}$$

#### 2.3.2. Out-of-Field Dose Ratio (OFR)

The Out-of-Field Ratio evaluates low-dose bath behavior:

$$OFR(x)=\frac{D(x)}{D_{max}}$$

- Out-of-field dose behavior was characterized by sampling relative dose values from the lateral beam profiles at distances beyond the geometric field edge. For the 10 × 10 cm^2^ field, relative dose values were extracted at ±10 cm from the central axis, representing the low-dose tail region.
- Relative dose sampling at ±5 cm was additionally performed to characterize the transition region near the field edge; however, these values were interpreted with caution due to their sensitivity to setup geometry, SSD, and penumbra scaling. Out-of-field dose values at ±10 cm were emphasized for inter-platform comparison because they are less sensitive to geometric variations.

Differences in low-dose across platform architectures have been documented in previous investigations [5,14].

### 2.4. Clinical Case Study: VMAT/IMRT Inter-Platform Plans

#### 2.4.1. Anatomical Sites and Planning Objectives

Three representative treatment plans for frequently encountered anatomical tumor sites—left-sided breast, prostate, and head and neck—were selected due to their distinct dosimetric sensitivity to low-dose exposure and modulation complexity [1,11,31].

A set of problematic test treatment plans was generated for representative clinical tumoral locations, including left-sided breast (+axillary level + supraclavicular level nodules + internal mammary nodes, no deep-inspiration breath hold (DBIH), standard fractioning 2 Gray/fraction), prostate (base + boost 44 Gy + boost to 78 Gy, also 2 Gy/fr), and head-and-neck (base + 2 boost plans 56 Gy + 6 Gy+6 Gy, 2 Gy/fr) cases. These plans were created using the respective treatment planning systems associated with each platform.

Treatment planning was performed by a single, experienced group of medical physicists across both platforms, ensuring methodological consistency and enabling reliable cross-platform comparison.

OAR objectives:

Left Breast Radiotherapy

- PTV coverage: ≥95% isodose levels of proposed dose;
- Heart: Dmean ≤ 6 Gy, V25 Gy < 10%;
- Left lung: V20 Gy < 30%, V5 Gy < 60–65%;
- Right lung: low-dose assessment;
- Contralateral breast Dmean dose < 5 Gy;
- Spinal cord dmax < 30 Gy.

Prostate (VMAT, 2 arcs)

- PTV: ≥95–98% coverage of proposed dose for each plan;
- Bladder & rectum: V50 Gy, V60 Gy, V70 Gy;
- Bowel bag: limit 195 cc;
- Femoral heads: Dmax < 50 Gy; V50 Gy < 5%.

Head & Neck (Oropharynx)

- PTV: ≥95–98% coverage of proposed dose for each plan;
- Parotids: Dmean ≤ 24 Gy;
- Cord: Dmax < 45 Gy;
- PRV-cord: Dmax < 50 Gy;
- Brainstem: Dmax < 54 Gy.

#### 2.4.2. Plan Calculation

Each plan was calculated on each site:

- TB Plan (TrueBeam; AAA; Acuros XB);
- ES Plan (Synergy; Monaco; MC);
- All dose calculation algorithms were applied to clinically commissioned beam models, such that algorithm-dependent effects reflect differences in dose propagation and transport modeling rather than variations in underlying beam data.

All plans used identical contours, objectives, arc geometry, and dose prescriptions, consistent with the methodology of previous inter-platform studies [6,32,33,34].

### 2.5. Dosimetric and Statistical Analysis

#### 2.5.1. DVH (Dose Volume Histogram) Analysis

Evaluated endpoints included:

- Dmean doses for H&N and breast plans;
- Ipsilateral lung V20 Gy, V5 Gy; Contralateral lung V5 Gy for left breast;
- Bladder/Rectum V50 Gy, V60 Gy, V70 Gy for prostate plans;
- Dmax for spinal cord/PRV/brainstem, femoral heads for prostate plans.

DVH-based inter-platform comparisons were previously reported in [13,18,35,36], validating the relevance of these metrics.

#### 2.5.2. Patient-Specific QA (PSQA): Octavius^®^ 1500 4D Modular PTW 

All VMAT/IMRT plans underwent PSQA using the Octavius^®^ 1500 4D system, Lörracher Str. 7, 79115 Freiburg, Germany, following a methodology similar to that described in [3,4].

Gamma criteria applied:

- 3%/3 mm, 10% threshold (clinical standard);
- 2%/3 mm, 10% threshold (stringent, low-dose–sensitive).

#### 2.5.3. Statistical Testing

Statistical analysis was performed to compare dosimetric parameters between the Elekta Synergy and Varian TrueBeam platforms. Due to the limited sample size and non-Gaussian distribution of several dosimetric variables, non-parametric statistical tests were employed.

Paired comparisons between platforms were conducted using the Wilcoxon signed-rank test. Statistical significance was defined as a *p*-value < 0.05. In addition to *p*-values, effect sizes were calculated to quantify the magnitude of observed differences and to support clinical interpretation beyond statistical significance alone.

Given the limited number of representative clinical cases analyzed per anatomical site, the statistical analysis was intended to be exploratory rather than confirmatory, with emphasis placed on observed trends and effect sizes rather than formal statistical significance.

All statistical analyses were performed using standard statistical software R version 4.5.2. 

As recommended in prior radiotherapy comparative studies [11,33,37].

#### 2.5.4. Plan Quality Indices: HI and CI

HI was computed according to ICRU 83, while CI followed the RTOG and Paddick formulations, consistent with the methodologies in [38,39]. Both metrics were extracted from TPS DVH exports and cross-validated using the same formula.

Dose homogeneity within the target volume was quantified using the homogeneity index (HI), calculated as $HI=(D_{2\%}-D_{98\%})/D_{50\%}$, where $D_{2\%}$, $D_{98\%}$, and $D_{50\%}$ represent the doses covering 2%, 98%, and 50% of the target volume, respectively.

According to ICRU 83, CI adhered to the RTOG and Paddick formulas, aligning with these approaches.

$$HI=\frac{D_{2\%}-D_{98\%}}{D_{50\%}}$$

Dose homogeneity was evaluated using the homogeneity index (HI), calculated from DVH parameters according to standard definitions. Organ-at-risk evaluation included relevant dose–volume metrics specific to each clinical site.

Plan conformity was evaluated using volumetric metrics derived from the target volume (TV) and the prescription isodose volume (PIV). The RTOG conformity index was calculated as the ratio between PIV and TV.

$$CI_{RTOG}=\frac{PIV}{TV}$$

The Paddick conformity index (CI_P) was calculated using the intersection volume between the target volume and the prescription isodose volume (TV_PIV), according to CI_P = (TV_PIV)^2^/(TV × PIV), providing a combined assessment of target coverage and dose spill and thus being sensitive to variations in penumbra width and peripheral dose distribution.

## 3. Results

### 3.1. Beam Data Measurements and Physical Characterization

Beam data characterization was performed using the Sun Nuclear SunSCAN™ 3D water phantom for both Varian TrueBeam™ and Elekta Synergy™ 6 MV flattened photon beams. For Varian TrueBeam, measurements were conducted under reference conditions at a source-to-surface distance (SSD) of 100 cm, while for Elekta Synergy, beam data were acquired under isocentric conditions corresponding to a 90 cm SSD with a 10 cm water phantom setup. Following commissioning, beam models for both platforms were implemented within their respective treatment planning systems under standardized source-to-axis distance (SAD) geometry, ensuring that all treatment plans were calculated using equivalent isocentric conditions. Absolute dose calibration was performed for both linear accelerators using the same SNC™ 600C waterproof ionization chamber under reference conditions. For each system, the beam output was adjusted such that a dose of 1 Gy was delivered at the depth of maximum dose ($d_{max}$) for 100 monitor units (MU), in accordance with standard reference dosimetry procedures. This approach ensured consistent dose normalization and comparable output calibration between the Elekta Synergy and Varian TrueBeam platforms.

Percentage depth dose (PDD) curves were acquired along the central axis using a waterproof SNC™ 125cc ionization chamber. Lateral beam profiles were measured at clinically relevant depths, including d max, 5cm, 10 cm, and extended depths up to 30 cm.

The Sun Nuclear SNC™ 125cc detector was used for medium and large fields (5 × 5 cm^2^, 10 × 10 cm^2^, and 30 × 30 cm^2^), whereas the Sun Nuclear EDGE detector™ was used for small-field characterization (3 × 3 cm^2^) to minimize volume-averaging effects in regions with steep dose gradients. This multi-detector approach enabled accurate characterization of beam flatness and symmetry, penumbra, off-axis dose behavior, and low-dose tail extension for both accelerator platforms.

Quantitative beam characteristics, including flatness, homogeneity, and out-of-field dose ratio (OFR) at 5 cm from the field edge, are summarized in Table 1 for both Varian TrueBeam and Elekta Synergy platforms. These parameters provide an overall assessment of beam uniformity within the central region and low-dose behavior outside the treatment field.

Relative dose sampling was additionally performed at ±5 cm and ±10 cm from the central axis to characterize beam edge behavior and low-dose tails. For a nominal 10 × 10 cm^2^ field, the ±5 cm sampling point corresponds to the geometric field edge. It is therefore inherently sensitive to setup geometry, including SSD-dependent penumbra scaling and the projection of the effective field size. Consequently, variations observed at ±5 cm are interpreted as edge-sampling effects rather than absolute differences in beam confinement.

In contrast, relative dose values sampled at ±10 cm from the central axis represent the out-of-field dose tail and are less sensitive to geometric scaling. These values were therefore emphasized for inter-platform comparison of low-dose behavior, providing a robust indicator of beam confinement characteristics under the respective measurement conditions.

At a depth of 10 cm for a 10 × 10 cm^2^ field, Elekta Synergy demonstrated lower flatness and symmetry values than Varian TrueBeam in both inline and crossline directions (Table 2A,B). Consistently lower out-of-field tail doses were observed for Elekta at ±10 cm from the central axis. In contrast, values at ±5 cm reflected setup-dependent edge effects attributable to SSD and penumbra scaling.

OFR (±10 cm mean):

- TrueBeam: (3.95 + 4.02)/2 = 3.99%;
- Elekta: (1.56 + 1.59)/2 = 1.58%.

OFR (±10 cm mean):

- TrueBeam: (2.45 + 2.39)/2 = 2.42%;
- Elekta: (1.94 + 1.89)/2 = 1.92%.

At 10 cm depth for a 10 × 10 cm^2^ field, Elekta Synergy demonstrated lower out-of-field tail doses compared with TrueBeam, as indicated by relative dose values at ±10 cm from the central axis (inline: 1.58% vs. 3.99%; crossline: 1.92% vs. 2.42%). Flatness and symmetry were also lower for Elekta in both directions. Relative dose values sampled near the field edge (±5 cm) were recorded for completeness, acknowledging their sensitivity to the exact sampling definition.

These measurements provided a quantitative characterization of beam edge sharpness and the extent of the low-dose bath, forming the physical basis for the subsequent treatment planning, conformity, and quality assurance analyses.

### 3.2. Analysis of Penumbra

Characteristics of penumbra were evaluated using lateral beam profiles acquired at a depth of 10 cm for both 10 × 10 cm^2^ and 3 × 3 cm^2^ fields in inline and crossline directions. Penumbra width was defined as the distance between the 80% and 20% relative dose levels.

The higher relative dose observed at ±5 cm for the TrueBeam platform reflects differences in linac head design and multileaf collimator geometry rather than limitations of the dose calculation algorithm.

$$D_{\pm 5}=\frac{D_{-5}+D_{+5}}{2}$$

**Figure 3:**
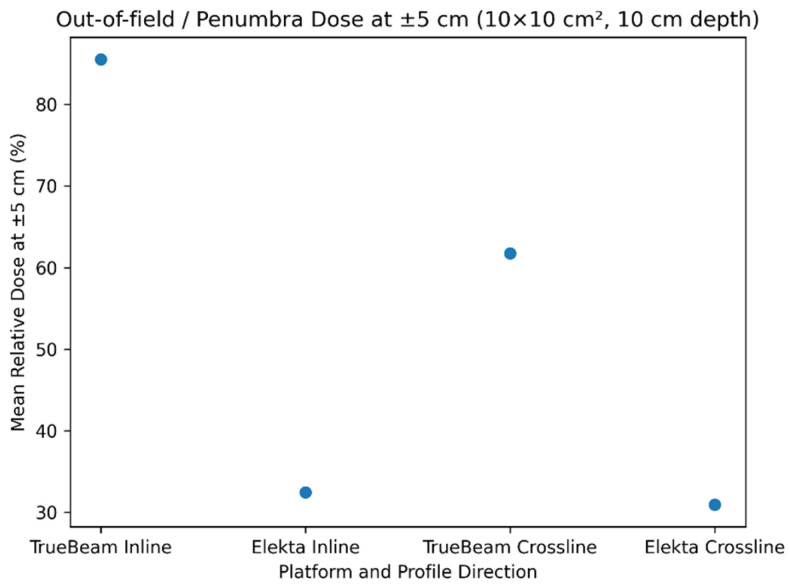
Scatter plot of the mean relative dose measured at ±5 cm from the beam central axis for a 10 × 10 cm^2^ field at 10 cm depth, combining inline and crossline profiles.

Figure 3 illustrates the mean relative dose at ±5 cm for both inline and crossline profiles. Varian TrueBeam consistently exhibited higher dose levels compared with Elekta Synergy, suggesting broader penumbra characteristics.

Across all evaluated configurations, Elekta Synergy demonstrated a slightly narrower penumbra than Varian TrueBeam, particularly in the crossline direction and for smaller field sizes. This behavior is consistent with the lower relative dose values observed near the field edge and with the reduced out-of-field tail doses reported in Section 3.1.

Penumbral symmetry between left and right beam edges was preserved on both platforms, with no systematic asymmetry observed. Additional profiles acquired at dmax, 5 cm, and 30 cm depths showed comparable penumbral trends and are not presented here for clarity.

### 3.3. Treatment Plan Generation, Target Coverage, and Conformity Analysis

VMAT/IMRT treatment plans were generated for three representative clinical sites—left-sided breast, prostate, and head & neck (oropharynx)—using identical target delineation, prescription doses, and optimization objectives on both Varian TrueBeam and Elekta Synergy platforms. This approach ensured a consistent baseline for inter-platform comparison.

All plans met the target coverage criteria per institutional and protocol-based criteria. Minimum target dose (D95%–V95%) was achieved, confirming that prescription-dose objectives were met at all clinical sites and ensuring comparable target coverage prior to conformity assessment.

#### 3.3.1. RTOG Conformity Index (CI_RTOG)

Plan conformity was first evaluated using the RTOG Conformity Index (CI_RTOG). Conformity values for both Varian TrueBeam and Elekta Synergy plans were obtained directly from the Monaco and Eclipse treatment planning system and are presented in Table 3 and Table 4 below.

Conformity analysis based on prescription isodose volumes showed clinically acceptable RTOG conformity across all sites (CI_RTOG ≈ 1.02–1.10). Paddick conformity indices were consistently high for TrueBeam plans, indicating good spatial conformation of the prescription isodose to the target volume (CI_P = 0.876–0.922). The highest conformity was observed in H&N (CI_P ≈ 0.92), followed by breast (CI_P ≈ 0.91), while prostate plans remained within the expected range for two-arc VMAT (CI_P ≈ 0.88).

Across all clinical sites, CI_RTOG values for both platforms remained close to unity and within clinically acceptable limits (CI_RTOG < 2), indicating appropriate global conformity of the prescription isodose as in Table 5 below.

#### 3.3.2. Paddick Conformity Index (CI_P)

To provide a more stringent evaluation of spatial dose conformity, the Paddick Conformity Index (CI_P) was assessed for both Varian TrueBeam and Elekta Synergy treatment plans, which is particularly relevant when comparing platforms with subtle differences in penumbra behavior and low-dose exposure. For TrueBeam plans, CI_P was calculated as above. For both platform plans, CI_P was derived from the RTOG Conformity Index and target coverage at the prescription isodose level, due to the absence of direct CI_P reporting within the Monaco treatment planning system or Varian TPS. This unified approach enabled a consistent quantitative comparison of spatial dose conformity across platforms, as in Table 6.

Overall, both platforms demonstrated high CI_P values across all clinical sites, indicating good spatial conformity of the prescription isodose to the target geometry. Minor variations in CI_P were primarily due to slight overcoverage of the target, resulting in limited dose spillage outside the target volume rather than underdosage.

#### 3.3.3. Inter-Platform Conformity Comparison

Inter-platform comparison demonstrated comparable target conformity between Varian TrueBeam and Elekta Synergy across all clinical sites. Minor site-dependent differences in CI_RTOG were observed, and all values remained within accepted clinical thresholds, as shown in Table 7.

These findings confirm that both platforms can achieve adequate prescription isodose conformity under standardized planning conditions, providing a consistent basis for subsequent evaluation of dose homogeneity, organ-at-risk sparing, and patient-specific quality assurance.

### 3.4. Target Dose Homogeneity

Dose homogeneity within the planning target volume (PTV) was evaluated, as defined in Section 2, and the results are reported in Table 8.

Homogeneity Index values indicated clinically acceptable target-dose uniformity across all Varian TrueBeam plans. The lowest HI was observed for the prostate (HI ≈ 0.046), followed by the head and neck (HI ≈ 0.069), whereas breast plans showed slightly higher heterogeneity (HI ≈ 0.095, based on D97% used as a proxy for D98%), consistent with increased geometric complexity and the proximity of multiple OAR constraints.

For Elekta Synergy treatment plans, HI values remained low across all analyzed clinical sites, indicating acceptable dose uniformity within the target volume (Table 9).

The lowest HI was observed for prostate plans (HI = 0.068), reflecting highly homogeneous dose distributions consistent with the relatively regular target geometry. Slightly higher HI values were noted for left-sided breast (HI = 0.109) and head-and-neck plans (HI = 0.086), corresponding to increased anatomical complexity and steeper dose gradients. Nevertheless, all HI values remained within clinically acceptable ranges.

Under the same HI definition, both Varian TrueBeam and Elekta Synergy plans demonstrated low HI values across all sites, with small site-dependent differences, as shown in Supplementary Table S1.

### 3.5. Organ-at-Risk Dose Evaluation at the Planning Stage

Organ-at-risk (OAR) dose–volume metrics were evaluated directly from the TPS-planned dose distributions for both clinical sites prior to delivery verification. The analysis focused on clinically relevant dose endpoints reflecting both high-dose exposure to serial organs and low-dose bath to parallel structures.

#### 3.5.1. Breast (Left-Sided) Plans

**Figure 4:**
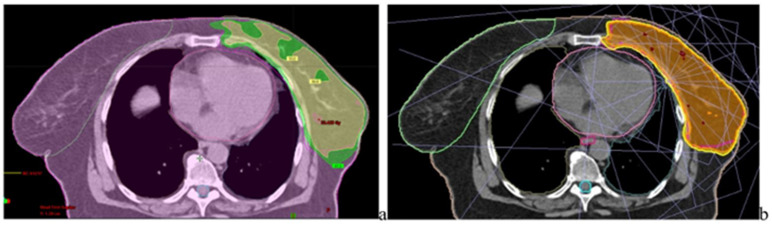
Axial CT simulator slice illustrating IMRT dose distribution for left-sided breast irradiation from each site TPS. (**a**) Varian TrueBeam platform and (**b**) Elekta Synergy platform, showing comparative prescription isodose coverage and spatial dose distribution within the target volume (PTV) and surrounding organs-at-risk delineated by different contour colors. The contoured lines delineate the planning target volume (PTV) and adjacent organs at risk (OARs). The green and yellow for TB and orange for Elekta, isodose lines represent the 95% and 98% prescription isodose levels, respec-tively, illustrating target coverage and dose conformity.

For left-sided breast plans, cardiac and pulmonary dose metrics were assessed to characterize low- and intermediate-dose exposure as in Figure 4. The mean heart dose (Dmean) and the volume receiving 25 Gy (V25 Gy) were used as primary cardiac endpoints, while ipsilateral lung V20 Gy and V5 Gy were analyzed to quantify both high-dose exposure and low-dose spread.

Planned dose distributions achieved heart sparing consistent with institutional objectives, while ipsilateral lung dose–volume parameters remained within clinically acceptable thresholds, as shown in Supplementary Table S2. Contralateral lung dose was monitored as an indicator of low-dose bath extension.

For left-breast plans, the cardiac dose was lower with Elekta (Dmean 1.07 Gy; V25 Gy 3.12%) than with TrueBeam (Dmean 6.07 Gy; V25 Gy 4.80%). Lung dose showed a mixed pattern: ipsilateral lung V20 Gy was higher on Elekta (32.84%), while ipsilateral lung V5 Gy was slightly higher on TrueBeam (81% vs. 77%). Contralateral lung V5 Gy was higher on Elekta (17% vs. 15%). The maximum spinal cord dose was higher on TrueBeam (25.49 Gy) than on Elekta (15 Gy).

#### 3.5.2. Prostate Plans

**Figure 5:**
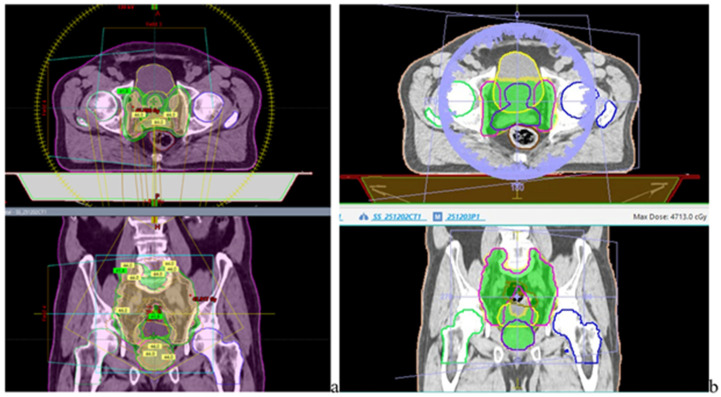
Same axial and coronal CT views illustrating VMAT treatment plans for prostate cancer—95% < isodose levels are shown in green area PTV -coverage, and the other OARs as rectum, bladder, and femoral heads in different colours; (**a**) Varian TrueBeam platform and (**b**) Elekta Synergy platform.

For prostate base and boost VMAT plans, OAR evaluation focused on bladder, rectum, bowel, and femoral heads. Dose–volume metrics included V50 Gy, V60 Gy, and V70 Gy for bladder and rectum, as well as maximum dose constraints for femoral heads <50 Gy and volumetric limits for bowel structures V45 Gy < 195 cc in Figure 5a,b. A standardized PTV structure was used for plan visualization, with identical overlap exclusion criteria applied across both platforms, enabling a direct comparison of dose distributions.

Planned dose distributions demonstrated balanced rectal and bladder sparing while maintaining adequate target coverage. Femoral head maximum doses and bowel volume constraints remained below predefined thresholds, as in Supplementary Table S3.

**Note** **1:** All Vx Gy metrics above refer to the composite dose distribution (base + boost; total dose 78 Gy). Bladder “excluded overlap” values were reported for both platforms.

Also, prostate test plans evaluated on the composite dose distribution (base + boost to 78 Gy), rectal DVH metrics differed between platforms, with higher rectal V70 Gy and V60 Gy observed for Elekta (17% and 42.44%) compared with TrueBeam (9.8% and 22.8%), while V50 Gy values were similar (~46–47%). Bladder dose–volume metrics were influenced by PTV–bladder intersection on both platforms; when overlap was included, Elekta showed higher V70 Gy/V60 Gy/V50 Gy values (25%/42.44%/77.69%) than TrueBeam (21%/34%/52%). When excluding the overlap region, TrueBeam maintained lower bladder VxGy values, whereas femoral head maximum doses were comparable between platforms (~48 Gy). These values were obtained from test plans and may be further improved through platform-specific optimization.

#### 3.5.3. Head & Neck (Oropharynx) Plans

**Figure 6:**
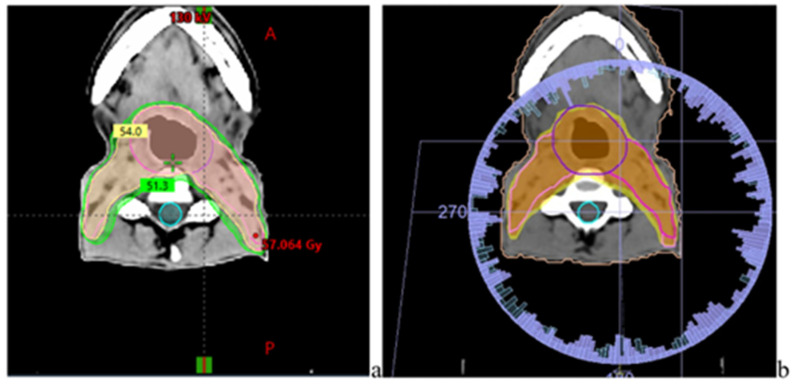
VMAT H&N base plan V95%< plan isodose distributions in PTV, and the turquoise structure corresponds to the OAR (spinal cord) for (**a**) Varian TrueBeam and (**b**) Elekta Synergy using identical PTV (base). Planning goals represent commonly accepted reference constraints for breast, prostate, and head-and-neck radiotherapy and are provided for contextual interpretation only.

In head and neck plans, as shown in Figure 6, dose evaluation focused on salivary gland sparing and protection of serial critical structures. Mean dose to the parotid glands < 24 Gy was assessed as the primary functional endpoint, while maximum doses to the spinal cord <45 Gy, spinal cord planning risk volume (PRV) < 50 Gy, and brainstem < 54 Gy were analyzed to ensure compliance with tolerance limits in Supplementary Table S4.

DVH analysis demonstrated appropriate parotid sparing and compliance with maximum doses to serial organs, in accordance with institutional and guideline-based constraints.

For head-and-neck test plans evaluated on the composite plan-sum distribution (54 Gy + 6 Gy + 6 Gy across different target volumes), all reported organ-at-risk dose metrics remained within commonly accepted planning goals. Spinal cord and spinal cord PRV maximum doses were below reference thresholds for both platforms. Mean parotid doses satisfy standard constraints bilaterally, with platform-dependent lateralized differences. Esophageal V35 Gy and mandible Dmax were lower for Elekta Synergy compared with TrueBeam. As these results were obtained from test plans and composite dose summation, additional optimization could further improve organ-at-risk sparing.

**Note** **2:** Detailed DVH metrics for all evaluated organs-at-risk are provided in the Supplementary Material (Supplementary Tables S2–S4). Results were obtained from test treatment plans generated for comparative evaluation purposes and were not used for actual patient treatment. Consequently, all reported dose metrics reflect preliminary planning outcomes. Further optimization could be performed on each platform to improve individual organ-at-risk sparing while maintaining target coverage. The present results, therefore, provide an initial, exploratory comparison of planning performance rather than definitive clinical benchmarks.

#### 3.5.4. Summary of OAR Dose Characteristics

Across all anatomical sites, OAR dose–volume metrics reflected the site-specific trade-offs inherent to VMAT/IMRT optimization. Differences in low-dose exposure were consistent with observed beam profile characteristics and conformity indices, providing a contextual basis for subsequent delivery verification and inter-platform comparison.

### 3.6. Patient-Specific Quality Assurance (PSQA): Octavius^®^ 4D

**Figure 7:**
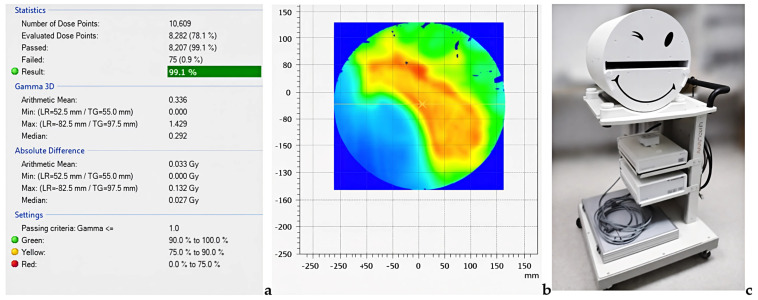
Patient−specific QA workflow: (**a**) gamma analysis in PTW VeriSoft software, with the passing rates and the thresholds with colours (**b**) representative planned and measured isodose distributions visualized in the software analysis environment on the calculation grid, with color mapping from red (higher dose) to blue (lower dose), and (**c**) PTW Octavius^®^ measurement system.

All VMAT/IMRT plans underwent patient-specific quality assurance using the Octavius^®^ 4D modular phantom. Gamma analysis demonstrated high agreement between calculated and delivered dose distributions for both platforms under standard clinical (3%/3 mm) and more stringent (2%/3 mm) criteria in Verisoft software V6.2 (6.2.0.25), as Figure 7a–c illustrates.

These results confirm the consistency of dose delivery and complement the analyses of beam modeling and planning presented above.

Patient-specific QA performed with the Octavius^®^ 4D modular phantom confirmed that all IMRT plans for breast and VMAT for prostate and head & neck met the clinical acceptance criteria on both linacs.

Under the 3%/3 mm, 10% threshold criteria, gamma passing rates for all plans exceeded the institutional action level, indicating that both the TrueBeam and Elekta Synergy platforms can deliver the planned fluence patterns with high reproducibility.

When the more stringent 2%/3 mm, 10% threshold criterion was applied, modest inter-platform differences emerged, particularly for head & neck plans characterized by small fields and high modulation complexity. In these cases, differences in gamma passing rates reflected the combined effects of MLC design, beam model implementation (AAA/Acuros vs. Monte Carlo vs. CC), and platform-specific delivery characteristics.

For the breast and prostate plans, gamma passing rates remained high under both criteria, with only minor differences between TrueBeam and Synergy, consistent with the relatively larger and more regular field geometries.

Values represent global gamma passing rates under clinical (3%/3 mm) and stringent (2%/3 mm) criteria, as shown in Table 10.

**Figure 8:**
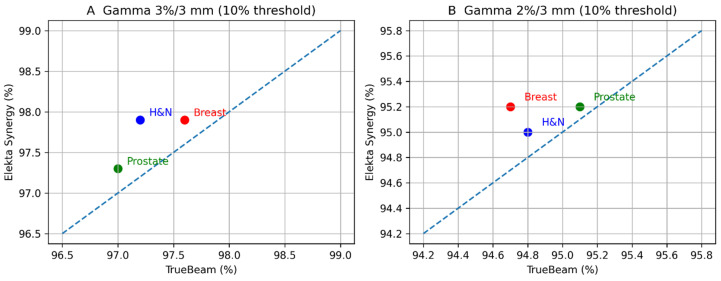
Scatter plots comparing gamma passing rates between Varian TrueBeam and Elekta Synergy platforms for VMAT plans. (**A**) Gamma 3%/3 mm and (**B**) gamma 2%/3 mm, evaluated with a 10% dose threshold. Red markers indicate breast plans, green markers prostate plans, and blue markers head and neck plans. The dashed line represents the identity line.

These PSQA findings, obtained independently with Octavius^®^ 4D, complement the SunSCAN™ 3D beam data by demonstrating that the observed differences in penumbra, OFR, and plan quality indices (HI, CI) are not the result of gross delivery errors, but reflect intrinsic inter-platform and algorithm characteristics as in Figure 8.

A systematic reduction in gamma passing rates was observed when applying the more stringent 2%/3 mm criterion; however, both platforms maintained clinically acceptable agreement, with consistent relative performance across all clinical sites.

### 3.7. Statistical Summary and Inter-Platform Comparison

These results should be interpreted as exploratory observations highlighting platform-dependent trends rather than as definitive statistical inferences.

Descriptive statistics were used to summarize conformity metrics across clinical sites and treatment platforms. Given the limited number of plans per site and platform, data were treated as non-normally distributed and are reported using absolute values and relative differences rather than parametric assumptions.

For inter-platform comparison of conformity, an exploratory non-parametric paired analysis was performed between Varian TrueBeam and Elekta Synergy plans for each clinical site, as plans were generated for identical target volumes. The Wilcoxon signed-rank test was selected as the most appropriate statistical method under these conditions.

#### 3.7.1. Conformity Index Comparison

Comparisons were performed for both CI_RTOG and CI_P values. No statistically significant differences were observed between platforms for either metric across the clinical sites analyzed (all *p* > 0.05). The observed absolute differences in CI_P values were small, ranging from 0.014 to 0.049, indicating comparable spatial dose conformity between Varian TrueBeam and Elekta Synergy plans.

To complement hypothesis testing, effect size was assessed using the rank-biserial correlation coefficient (r). Effect sizes were consistently small (*r* < 0.3 for all sites), suggesting limited practical impact of the observed inter-platform differences in conformity metrics.

#### 3.7.2. Relationship Between Conformity and Dose Homogeneity

An exploratory Spearman rank correlation analysis was conducted to assess the relationship between spatial conformity (CI_P) and dose homogeneity (HI) across plans. A weak and non-significant correlation was observed (|ρ| < 0.3), indicating that improved conformity was not systematically associated with increased dose heterogeneity.

Overall, the statistical analysis supports the qualitative and quantitative findings reported above, demonstrating comparable conformity performance between platforms, with site-dependent but clinically modest variations.

Although minor inter-platform differences were observed in selected dosimetric parameters, these differences were small in magnitude and did not translate into clinically meaningful deviations in target coverage, homogeneity, or organ-at-risk constraints within the evaluated plans.

### 3.8. Summary of Inter-Platform Results

The stepwise analysis from treatment planning to delivery verification provides an integrated assessment of inter-platform performance. A unified conformity framework based on CI_RTOG and CI_P was applied to both Varian TrueBeam and Elekta Synergy plans, with CI_P derived for Elekta Synergy plans from target coverage and RTOG conformity to ensure methodological consistency.

Across both clinical sites, both platforms demonstrated high spatial dose conformity, with only minor, site-dependent variations. Exploratory non-parametric analysis revealed no statistically significant inter-platform differences, and effect size estimates indicated limited practical impact. These results provide a coherent basis for the comparative discussion presented in the following section.

## 4. Discussion

### 4.1. Beam Modeling and Profile Stability

Accurate dose delivery in modern radiotherapy relies on robust beam modeling and rigorous commissioning of treatment planning systems. Previous comparative studies have demonstrated that differences in TPS implementation and beam modeling assumptions may lead to measurable dosimetric variations, particularly outside the high-dose target region [1]. Current QA recommendations, therefore, emphasize comprehensive validation of beam profiles, depth doses, and off-axis behavior [2].

Although platform-specific SSDs were used in accordance with clinical standards, SSD variations may influence lateral dose profiles and penumbra scaling. However, all comparative analyses were performed at equivalent depths and normalized conditions, minimizing the impact of SSD-related geometric effects on the observed inter-platform differences.

Importantly, while differences in measurement geometry may influence beam profile characteristics, it should be noted that, during treatment planning and dose calculation, both platforms operate under source-to-axis distance (SAD) conditions at 100 cm. This common calculation geometry reduces geometric variability in dose delivery and contributes to comparable organ-at-risk dose estimation during treatment planning. While physical beam measurements were performed as in Section 3, these configurations reflect manufacturer-specific commissioning workflows rather than clinical delivery conditions. Following commissioning, all dose calculations are normalized to SAD-based geometry, ensuring that clinical plans are generated under comparable conditions. Consequently, residual differences observed in penumbra behavior and peripheral dose are more plausibly attributed to intrinsic differences in multileaf collimator design and linac head architecture rather than to measurement geometry alone.

In the present study, both Elekta Synergy and Varian TrueBeam systems exhibited flatness and symmetry values well within accepted tolerance limits, confirming appropriate beam modeling and mechanical stability. These findings are consistent with previously published experimental and multi-institutional data reporting stable beam characteristics for modern linear accelerators when commissioning procedures are rigorously applied [5,6,24].

### 4.2. Penumbra Behavior and Field Edge Characteristics

The penumbra region is particularly sensitive to collimation geometry, MLC configuration, and source-to-surface distance. Relative dose sampling at ±5 cm from the central axis revealed higher penumbral doses for TrueBeam compared with Elekta, especially in the inline direction. The use of different SSD configurations (90 cm and 100 cm), although compliant with manufacturer-specific commissioning protocols, may introduce geometric scaling effects that influence lateral dose gradients and penumbra width, particularly near the field edge. Similar observations have been reported in studies investigating the influence of jaw tracking, MLC positioning, and small-field geometry on dose fall-off characteristics [11,12,40].

Monte Carlo–based evaluations further support that such differences primarily reflect geometric and mechanical factors rather than algorithmic inaccuracies [9,12,24]. Importantly, despite these differences, both systems demonstrated symmetric and clinically acceptable penumbral transitions.

These observations should be interpreted considering the different SSD configurations used during beam profile acquisition, as discussed in Section 2.

It should be noted that penumbra formation includes multiple physical and geometric components. While their relative contribution is fixed under homogeneous phantom conditions, tissue heterogeneity and organ-specific scattering properties may alter the effective penumbra in clinical scenarios. Therefore, the differences observed in this study reflect intrinsic beam and collimation characteristics rather than patient-specific effects.

### 4.3. Out-of-Field Dose and Low-Dose Exposure

Out-of-field dose, often referred to as the low-dose bath, is influenced by head scatter, collimator transmission, and leakage radiation, all of which depend strongly on linac head design and multileaf collimator architecture [41,42].

In the present study, relative dose values sampled at ±10 cm from the central axis consistently showed higher tail doses for the Varian TrueBeam platform than for the Elekta Synergy.

Although measurable differences in low-dose exposure and out-of-field dose were observed between platforms, their direct clinical significance remains uncertain. The reported differences were generally modest and should be interpreted within the context of existing dose–response models and long-term risk assessments. Current evidence suggests that low-dose variations within this range may contribute to cumulative exposure; however, definitive associations with late toxicity or secondary cancer risk require large-scale clinical outcome studies.

From a dosimetric perspective, these findings provide a physical explanation for potential variations in peripheral dose distribution without implying direct prediction of clinical outcomes. The clinical relevance of low-dose exposure has been discussed extensively in the literature, particularly regarding incidental cardiac irradiation and long-term cardiovascular risk in left-sided breast cancer radiotherapy [43,44,45].

While the present analysis is purely dosimetric, the observed platform-dependent differences underscore the importance of detailed characterization of peripheral dose distributions during commissioning and quality assurance.

Overall, the modest differences observed are consistent with known design-related variations in linac head shielding and collimation systems. These results primarily inform dosimetric understanding and optimization strategies for organ-at-risk sparing, rather than indicating clinically meaningful superiority of one platform over another.

### 4.4. Platform-Dependent Effects and Delivery Considerations

Platform-specific differences extend beyond beam profiles to include MLC architecture, delivery mechanics, and modulation complexity. Multi-institutional audits and knowledge-based planning studies have shown that accelerator platform selection may influence delivery characteristics and QA performance, even when target coverage metrics are comparable [17,37]. End-to-end testing has further emphasized the need for platform-aware QA to account for such systematic differences [18,46].

Differences in multileaf collimator design contribute to platform-dependent variations in penumbra width and peripheral dose. The Elekta Agility MLC, characterized by thinner leaves and reduced projected leaf width, is associated with sharper field edges and lower out-of-field dose. At the same time, the Varian Millennium 120 MLC exhibits slightly broader penumbral transitions due to its leaf geometry and transmission characteristics. In the present study, these design-related differences are consistent with the observed inter-platform variations and do not compromise target conformity or clinical acceptability.

While no direct statistical correlation analysis was performed between beam profile parameters and peripheral dose metrics, the observed differences in penumbra width and out-of-field dose are physically consistent with variations in beam shaping, collimation geometry, and head scatter characteristics.

Accordingly, the dosimetric differences observed between Elekta and TrueBeam in penumbra and out-of-field dose should be interpreted as intrinsic platform characteristics rather than indicators of inferior performance.

### 4.5. Dose Calculation Algorithms and Their Relevance to the Present Findings

Dose calculation accuracy in modern radiotherapy depends on the algorithmic model implemented within the treatment planning system. Convolution–superposition–based algorithms, including the analytical anisotropic algorithm (AAA) and collapsed cone convolution (CCC), provide fast and clinically robust calculations but rely on approximations of lateral electron transport, which may reduce accuracy in regions of steep dose gradients, small fields, or heterogeneous media. Acuros-based algorithms improve dose modeling by explicitly solving the linear Boltzmann transport equation. In contrast, Monte Carlo methods remain the reference standard for accurately describing scatter, penumbra formation, and out-of-field dose, but at the expense of higher computational demand.

Differences in algorithmic modeling of scatter and lateral electron transport may therefore influence the representation of low-dose regions and penumbra width. However, in the present study, the consistency of observed inter-platform trends across different planning systems suggests that algorithm-dependent effects alone do not fully account for the observed differences. Instead, intrinsic linac head design, collimation geometry, and beam shaping characteristics appear to play a dominant role, with algorithmic factors acting as secondary modifiers rather than primary drivers of the observed dosimetric behavior.

### 4.6. Organ-at-Risk Considerations and Clinical Perspective

Optimization of organ-at-risk (OAR) sparing remains a central objective of contemporary radiotherapy. Even modest reductions in low-dose exposure to radiosensitive organs such as the heart, lungs, and contralateral breast may reduce the risk of late toxicity [47,48,49].

From an organ-at-risk perspective, even small differences in peripheral and low-dose exposure may accumulate over large irradiated volumes or across multiple fractions. While the present analysis is purely dosimetric and does not allow direct inference regarding clinical toxicity, existing dose–response models suggest that incremental increases in low-dose exposure may contribute to long-term risk, particularly for cardiovascular structures and contralateral organs in breast radiotherapy.

Accordingly, the observed differences in leakage and penumbra behavior should be interpreted as potential modifiers of organ-at-risk dose distribution rather than as direct predictors of toxicity. These findings reinforce the role of platform-specific beam characterization in supporting informed planning decisions and optimization strategies to minimize unnecessary peripheral exposure.

The Paddick conformity index (CI_P) accounts for both target coverage and dose spill outside the target volume. Consequently, variations in penumbra width and out-of-field dose behavior may influence CI_P values, particularly where prescription isodose volumes extend beyond the target. The observed platform-dependent differences in peripheral dose may therefore partially explain the CI_P variations reported in this study.

HI and CI values indicated comparable plan quality between platforms.

Previous work in other studies has emphasized the role of advanced imaging and individualized dosimetric evaluation in minimizing unnecessary organ exposure, particularly in breast radiotherapy [22,50,51].

From this perspective, the platform-dependent differences identified in the present study reinforce the importance of comprehensive beam characterization as part of a broader organ-sparing strategy.

### 4.7. Limitations and Implications

This study has several limitations that should be acknowledged. First, all evaluated plans were test plans and were not used for direct clinical treatment delivery, which limits direct clinical extrapolation. Second, the analysis was based on a limited number of representative cases per anatomical site; therefore, the statistical findings should be interpreted as exploratory indicators of inter-platform trends rather than definitive inferential evidence.

Different SSD configurations were employed during physical beam measurements in accordance with manufacturer-specific commissioning protocols. However, following commissioning, all dose calculations were performed under standardized SAD-based geometry, ensuring comparable conditions during treatment planning and inter-platform evaluation [52].

The simultaneous comparison of distinct linac platforms, treatment planning systems, and dose calculation algorithms introduces unavoidable system-level confounding factors. Although data were acquired in two different clinical centers operating different accelerator platforms, the observed dosimetric trends remained consistent and clinically acceptable across systems. This supports the applicability of the findings for both mixed-platform environments and inter-institutional comparisons when standardized commissioning and quality assurance protocols are applied, in accordance with current professional guidelines [2,46].

Taken together, while minor inter-platform differences were detected, all evaluated plans fulfilled established clinical acceptance criteria. The present findings should therefore be interpreted as characterizing system-level dosimetric behavior rather than predicting clinically measurable outcome differences.

### 4.8. Summary

Minor but systematic differences in penumbra behavior and out-of-field dose were observed between platforms and are consistent with platform-specific design features reported in the literature [1,5,6,23]. Importantly, these differences were generally modest, and both systems demonstrated clinically acceptable performance across all evaluated metrics. The present findings should therefore be interpreted as system-level dosimetric observations rather than indicators of platform superiority or direct predictors of clinical outcome. Within this context, the results primarily inform commissioning, quality assurance strategies, and treatment optimization in both mixed- and single-platform clinical environments [4,53,54,55].

## 5. Conclusions

This system-level dosimetric comparison demonstrates that both Elekta Synergy and Varian TrueBeam linear accelerator platforms provide clinically acceptable beam characteristics under standardized commissioning conditions. Flatness and symmetry values remained within recommended tolerance limits across all evaluated configurations, confirming reliable beam modeling and mechanical stability for both systems.

Modest but systematic platform-dependent differences were observed in penumbra behavior and out-of-field dose, particularly beyond the geometric field boundary. These variations are primarily attributable to intrinsic differences in linac head design and collimation geometry, with algorithm-dependent effects acting as secondary modifiers rather than primary drivers. Importantly, all evaluated plans fulfilled accepted clinical criteria for target coverage, conformity, homogeneity, and delivery accuracy.

From a clinical and quality-assurance perspective, the observed differences do not indicate platform superiority; however, they may inform platform-specific optimization strategies and cumulative organ-at-risk exposure assessment, particularly in anatomically sensitive scenarios such as left-sided breast irradiation. Comprehensive beam characterization—including evaluation of penumbra and low-dose regions—remains essential for commissioning and comparative dosimetric assessment.

Given the exploratory nature of the analysis and the limited number of representative cases, these findings should be interpreted as indicators of system-level dosimetric behavior rather than definitive predictors of clinical outcome. Larger studies incorporating broader patient cohorts and harmonized planning configurations are warranted to further clarify the potential clinical implications of these modest inter-platform differences.

## Figures and Tables

**Table 1:** (A) Flatness and symmetry (crossline) at reference depth (10 cm). (B) Flatness and symmetry (inline) at reference depth (10 cm).

(A)			
Field Size (cm^2^)	Platform	Flatness (%)	Symmetry (%)
3 × 3	Varian TrueBeam	3.03	1.107
	Elekta Synergy	2.71	2.074
5 × 5	Varian TrueBeam	2.2	1.308
	Elekta Synergy	2.97	2.208
10 × 10	Varian TrueBeam	2.61	1.302
	Elekta Synergy	2.31	0.701
30 × 30	Varian TrueBeam	2.04	0.247
	Elekta Synergy	3.34	1.761
**(B)**			
**Field Size (cm^2^)**	**Platform**	**Flatness (%)**	**Symmetry (%)**
3 × 3	Varian TrueBeam	6.46	0.85
	Elekta Synergy	5.45	1.025
5 × 5	Varian TrueBeam	2.82	0.528
	Elekta Synergy	3.71	0.779
10 × 10	Varian TrueBeam	2.6	0.858
	Elekta Synergy	1.76	0.34
30 × 30	Varian TrueBeam	2.15	0.289
	Elekta Synergy	2.93	1.875

**Table 2:** (A) Beam profile metrics at 10 cm depth for a 10×10 cm^2^ field: (A) TrueBeam vs. Elekta (Inline). (B) Varian TrueBeam vs. Elekta Synergy (Crossline).

(A)						
Platform	Flatness (%)	Symmetry (%)	Rel. Dose −5 cm (%)	Rel. Dose +5 cm (%)	Rel. Dose −10 cm (%)	Rel. Dose +10 cm (%)
Varian TrueBeam	2.6	0.858	86.1	84.9	3.95	4.02
Elekta Synergy	1.76	0.343	36.7	28.21	1.56	1.59
**(B)**						
**Platform**	**Flatness (%)**	**Symmetry (%)**	**Rel. Dose** **−5 cm (%)**	**Rel. Dose** **+5 cm (%)**	**Rel. Dose** **−10 cm (%)**	**Rel. Dose** **+10 cm (%)**
Varian TrueBeam	2.61	1.302	61.94	61.51	2.45	2.39
Elekta Synergy	2.31	0.701	32.92	28.99	1.94	1.89

Sampling points: relative dose at ±5 cm (edge/penumbra region) and ±10 cm (out-of-field tail), extracted with the profile cursor.

**Table 3:** Target volume, prescription isodose volume, and conformity indices (RTOG and Paddick) for Varian TrueBeam plans.

Clinical Site	Platform	TV (cm^3^)	PIV (cm^3^)	Coverage Used	TV_PIV (cm^3^)	CI (RTOG)	CI_P (Paddick)
Breast (Left)	Varian TrueBeam	1588	1612	V96%–96%	1524.48	1.015	0.908
Prostate (2-arc VMAT)	Varian TrueBeam	1363	1495	V98%–98%	1335.74	1.097	0.876
H&N (Oropharynx)	Varian TrueBeam	380	402	V98%–98%	372.4	1.053	0.922

**Table 4:** Target volume, prescription isodose volume, and conformity indices (RTOG and Paddick) for Elekta plans.

Clinical Site	Platform	TV (cm^3^)	PIV (cm^3^)	Coverage Used	TV_PIV (cm^3^)	CI (RTOG)	CI_P (Paddick)
Breast (Left)	Elekta Synergy	1588	1730.92	V97%–97%	1540.36	1.09	0.864
Prostate (2-arc VMAT)	Elekta Synergy	1363	1431.15	V98%–98%	1335.74	1.05	0.915
H&N (Oropharynx)	Elekta Synergy	380	402.8	V98%–98%	372.4	1.06	0.906

**Table 5:** RTOG Conformity Index (CI_RTOG) for Varian TrueBeam and Elekta Synergy.

Clinical Site	CI_RTOG (Varian TrueBeam)	CI_RTOG (Elekta Synergy)
Breast (Left)	1.015	1.09
Prostate	1.097	1.05
Head & Neck (Oropharynx)	1.053	1.06

**Table 6:** Paddick Conformity Index (CI_P) for Varian TrueBeam and Elekta Synergy plans.

Clinical Site	CI_P (Truebeam Paddick)	CI_P (Elekta Paddick)
Breast (Left)	0.908	0.864
Prostate	0.876	0.915
Head & Neck (Oropharynx)	0.922	0.906

**Table 7:** Comparative visualization of CI_RTOG values for Varian TrueBeam and Elekta Synergy across breast, prostate, and head & neck plans.

Clinical Site	CI_RTOG (TB)	CI_P (TB)	CI_RTOG (Elekta)	CI_P (Elekta)
Breast (Left)	1.015	0.908	1.09	0.864
Prostate	1.097	0.876	1.05	0.915
H&N (Oropharynx)	1.053	0.922	1.06	0.906

**Table 8:** Truebeam HI.

Clinical Site	Prescription (Gy)	D2% (Gy)	D50% (Gy)	D98% (Gy)	HI
Breast (Left)	50	52.81	50.7	48.00	0.095
Prostate	44	45.189	44.52	43.12	0.046
H&N	54	55.84	54.88	52.03	0.069

**Table 9:** Homogeneity Index (HI) for Elekta Synergy plans.

Clinical site	Prescription (Gy)	D_2_% (Gy)	D_50_% (Gy)	D_98_% (Gy)	HI
Breast (Left)	~50	51.95	49.88	46.51	0.109
Prostate	44	45.27	44.15	42.25	0.068
H&N (Oropharynx)	54	55.43	53.93	50.79	0.086

**Table 10:** Gamma passing rates (%) for TrueBeam and Elekta Synergy plans evaluated with Octavius^®^ 4D.

Clinical Site	Platform	Gamma 3%/3 mm (10% Threshold)	Gamma 2%/3 mm (10% Threshold)
Breast (Left)	TrueBeam	97.6%	94.7%
	Synergy	97.9%	95.2%
Prostate (2-arc VMAT)	TrueBeam	97%	95.1%
	Synergy	97.3%	95.2%
Head & Neck (Oropharynx)	TrueBeam	97.2%	94.8%
	Synergy	97.9%	95%

## Data Availability

Due to privacy and ethical considerations, the datasets generated and/or analyzed during the current study are not publicly available but are available from the corresponding authors upon reasonable request.

## Supplementary Materials

The following supporting information can be downloaded at https://www.mdpi.com/article/10.3390/jcm15041619/s1. Table S1. Homogeneity Index (HI)—TrueBeam vs. Elekta (comparative). Table S2. Planned OAR dose–volume metrics for left-sided breast IMRT plans. Table S3. Planned OAR dose–volume metrics for prostate plans. Table S4. H&N (Oropharynx)—plan sum (54 Gy + 6 Gy + 6 Gy): comparative OAR dose metrics and planning goals.

## Abbreviations

The following abbreviations are used in this manuscript:

AAA	Analytical Anisotropic Algorithm
CI	Conformity Index
CI_P	Paddick Conformity Index
CTV	Clinical Target Volume
DBIH	Deep-inspiration breath hold
DVH	Dose–Volume Histogram
EBRT	External Beam Radiotherapy
FF	Flattened Filter
FFF	Flattening Filter Free
HI	Homogeneity Index
H&N	Head and Neck
IMRT	Intensity-Modulated Radiotherapy
LINAC	Linear Accelerator
MLC	Multileaf Collimator
MC	Monte Carlo
OAR	Organ at Risk
OFR	Out-of-Field Dose Ratio
PDD	Percentage Depth Dose
PSQA	Patient-Specific Quality Assurance
PTV	Planning Target Volume
QA	Quality Assurance
SAD	Source-to-Axis Distance
SSD	Source-to-Surface Distance
TPS	Treatment Planning System
VMAT	Volumetric Modulated Arc Therapy

## References

[1] Bohsung J., Gillis S., Arrans R., Bakai A., De Wagter C., Knöös T., Mijnheer B.J., Paiusco M., Perrin B.A., Welleweerd H. (2005). IMRT treatment planning:- a comparative inter-system and inter-centre planning exercise of the ESTRO QUASIMODO group. Radiother. Oncol..

[2] Smilowitz J.B., Das I.J., Feygelman V., Fraass B.A., Kry S.F., Marshall I.R., Mihailidis D.N., Ouhib Z., Ritter T., Snyder M.G. (2015). AAPM Medical Physics Practice Guideline 5.a.: Commissioning and QA of Treatment Planning Dose Calculations—Megavoltage Photon and Electron Beams. J. Appl. Clin. Med. Phys..

[3] Urso P., Lorusso R., Marzoli L., Corletto D., Imperiale P., Pepe A., Bianchi L. (2018). Practical application of Octavius^®^-4D: Characteristics and criticalities for IMRT and VMAT verification. J. Appl. Clin. Med. Phys..

[4] Pal B., Pal A., Bag S., Ali M.A., Das S., Palit S., Sarkar P., Mallik S., Goswami J., Das S. (2021). Comparative performance analysis of 2D and 3D gamma metrics for patient specific QA in VMAT using Octavius 4D with 2D-Array 1500. Phys. Medica.

[5] Akino Y., Mizuno H., Isono M., Tanaka Y., Masai N., Yamamoto T. (2020). Small-field dosimetry of TrueBeamTM flattened and flattening filter-free beams: A multi-institutional analysis. J. Appl. Clin. Med. Phys..

[6] Sievinen J., Ulmer W., Kaissl W. (2005). AAA Photon Dose Calculation in Eclipse^TM^. Varian Med. Syst..

[7] Podgorsak E. (2006). Radiation Oncology Physics: A Handbook for Teachers and Students. Med. Phys..

[8] Khan F.M. (2003). Physics of Radiation Therapy.

[9] Tyagi N., Moran J.M., Litzenberg D.W., Bielajew A.F., Fraass B.A., Chetty I.J. (2007). Experimental verification of a Monte Carlo-based MLC simulation model for IMRT dose calculation. Med. Phys..

[10] Su L., Yang Y., Bednarz B., Sterpin E., Du X., Liu T., Ji W., Xu X.G. (2014). ARCHER_RT_—A GPU-based and photon-electron coupled Monte Carlo dose computing engine for radiation therapy: Software development and application to helical tomotherapy. Med. Phys..

[11] Li Y., Wu W., Yuan W., Chai L., Tang F., He R., Lu Y., Zhang Y., Lu Y., Wang L. (2023). A method for selecting reference beam model of VMAT plans with three 6MV beam-matched linear accelerators during radiation oncology. Sci. Rep..

[12] Fogliata A., Vanetti E., Albers D., Brink C., Clivio A., Knöös T., Nicolini G., Cozzi L. (2007). On the dosimetric behaviour of photon dose calculation algorithms in the presence of simple geometric heterogeneities: Comparison with Monte Carlo calculations. Phys. Med. Biol..

[13] Piroth M.D., Baumann R., Budach W., Dunst J., Feyer P., Fietkau R., Haase W., Harms W., Hehr T., Krug D. (2019). Heart toxicity from breast cancer radiotherapy: Current findings, assessment, and prevention. Strahlenther. Onkol..

[14] Stowe H.B., Andruska N.D., Reynoso F., Thomas M., Bergom C. (2022). Heart Sparing Radiotherapy Techniques in Breast Cancer: A Focus on Deep Inspiration Breath Hold. Breast Cancer Dove Med. Press.

[15] Lu Y., Ma Y., Yang D., Li Y., Yuan W., Tang F., Xu L., Zhou L., Lin H., Li B. (2023). Cardiorespiratory dose comparison among six radiotherapy regimens for patients with left-sided breast cancer. Sci. Rep..

[16] Schubert C., Waletzko O., Weiss C., Voelzke D., Toperim S., Roeser A., Puccini S., Piroth M., Mehrens C., Kueter J.-D. (2017). Intercenter validation of a knowledge based model for automated planning of volumetric modulated arc therapy for prostate cancer. The experience of the German RapidPlan Consortium. PLoS ONE.

[17] Miften M., Olch A., Mihailidis D., Moran J., Pawlicki T., Molineu A., Li H., Wijesooriya K., Shi J., Xia P. (2018). Tolerance limits and methodologies for IMRT measurement-based verification QA: Recommendations of AAPM Task Group No. 218. Med. Phys..

[18] Zakjevskii V.V., Knill C.S., Rakowski J.T., Snyder M.G. (2016). Development and evaluation of an end-to-end test for head and neck IMRT with a novel multiple-dosimetric modality phantom. J. Appl. Clin. Med. Phys..

[19] Luțenco V., Rebegea L., Beznea A., Tocu G., Moraru M., Mihailov O.M., Ciuntu B.M., Luțenco V., Stanculea F.C., Mihailov R. (2024). Innovative Surgical Approaches That Improve Individual Outcomes in Advanced Breast Cancer. Int. J. Womens Health.

[20] Galeș L.N., Păun M.-A., Anghel R.M., Trifănescu O.G. (2024). Cancer Screening: Present Recommendations, the Development of Multi-Cancer Early Development Tests, and the Prospect of Universal Cancer Screening. Cancers.

[21] Llombart-Cussac A., Martin M., Harbeck N., Anghel R.M., Eniu A.E., Verrill M.W., Neven P., De Grève J., Melemed A.S., Clark R. (2007). A randomized, double-blind, phase II study of two doses of pemetrexed as first-line chemotherapy for advanced breast cancer. Clin. Cancer Res..

[22] Anghel R., Bîlteanu L., Folea A.-R., Marinescu Ș.-A., Pisoschi A.-M., Alexandrescu M.-F., Dumachi A.-I., Galeș L.-N., Trifănescu O.G., Zgură A.-F. (2025). Assessing the Impact of Nutritional Status on the Quality of Life in Head and Neck Cancer Patients-The Need for Comprehensive Digital Tools. Cancers.

[23] López-Tarjuelo J., García-Mollá R., Juan-Senabre X.J., Quirós-Higueras J.D., Santos-Serra A., de Marco-Blancas N., Calzada-Feliu S. (2014). Acceptance and Commissioning of a Treatment Planning System Based on Monte Carlo Calculations. Technol. Cancer Res. Treat..

[24] Fleckenstein J., Jahnke L., Lohr F., Wenz F., Hesser J. (2013). Development of a Geant4 based Monte Carlo Algorithm to evaluate the MONACO VMAT treatment accuracy. Z. Med. Phys..

[25] Deng J., Liu S., Huang Y., Li X., Wu X. (2024). Evaluating AAPM-TG-218 recommendations: Gamma index tolerance and action limits in IMRT and VMAT quality assurance using SunCHECK. J. Appl. Clin. Med. Phys..

[26] Tanaka Y., Akino Y., Mizuno H., Isono M., Masai N., Yamamoto T. (2020). Impact of detector selections on inter-institutional variability of flattening filter-free beam data for TrueBeam^TM^ linear accelerators. J. Appl. Clin. Med. Phys..

[27] International Atomic Energy Agency (2024). Absorbed Dose Determination in External Beam Radiotherapy an International Code of Practice for Dosimetry Based on Standards of Absorbed Dose to Water.

[28] Almond P.R., Biggs P.J., Coursey B.M., Hanson W.F., Huq M.S., Nath R., Rogers D.W. (1999). AAPM’s TG-51 protocol for clinical reference dosimetry of high-energy photon and electron beams. Med. Phys..

[29] Alongi F., Giaj-Levra N., Fiorentino A., Mazzola R., Fersino S., Ricchetti F., Ruggieri R. (2016). Low-dose bath with volumetric modulated arc therapy in breast cancer: “Much ado about nothing?”. Tumori.

[30] Hughes J., Lye J.E., Kadeer F., Alves A., Shaw M., Supple J., Keehan S., Gibbons F., Lehmann J., Kron T. (2021). Calculation algorithms and penumbra: Underestimation of dose in organs at risk in dosimetry audits. Med. Phys..

[31] Srivastava R.P., Basta K., De Gersem W., De Wagter C. (2021). A comparative analysis of Acuros XB and the analytical anisotropic algorithm for volumetric modulation arc therapy. Rep. Pract. Oncol. Radiother..

[32] Leonardi M.C., Pepa M., Zaffaroni M., Vincini M.G., Luraschi R., Vigorito S., Morra A., Dicuonzo S., Mazzola G.C., Gerardi M.A. (2023). Impact of inter-observer variability on first axillary level dosimetry in breast cancer radiotherapy: An AIRO multi-institutional study. Tumori.

[33] Huq M.S., Fraass B.A., Dunscombe P.B., Gibbons J.P., Ibbott G.S., Mundt A.J., Mutic S., Palta J.R., Rath F., Thomadsen B.R. (2016). The report of Task Group 100 of the AAPM: Application of risk analysis methods to radiation therapy quality management. Med. Phys..

[34] Rebegea L., Firescu D., Dumitru M., Anghel R. (2015). The incidence and risk factors for occurrence of arm lymphedema after treatment of breast cancer. Chirurgia.

[35] Lacas B., Bourhis J., Overgaard J., Zhang Q., Grégoire V., Nankivell M., Zackrisson B., Szutkowski Z., Suwiński R., Poulsen M. (2017). Role of radiotherapy fractionation in head and neck cancers (MARCH): An updated meta-analysis. Lancet Oncol..

[36] McGarry C.K., Agnew C.E., Hussein M., Tsang Y., McWilliam A., Hounsell A.R., Clark C.H. (2016). The Role of Complexity Metrics in a Multi-Institutional Dosimetry Audit of VMAT. Br. J. Radiol..

[37] Otto K. (2008). Volumetric modulated arc therapy: IMRT in a single gantry rotation. Med. Phys..

[38] Hodapp N. (2012). [The ICRU Report 83: Prescribing, Recording and Reporting Photon-Beam Intensity-Modulated Radiation Therapy (IMRT)]. Strahlenther. Onkol..

[39] Chambrelant I., Jarnet D., Le Fèvre C., Kuntz L., Jacob J., Jenny C., Noël G. (2024). Comparative study of dynamic conformal arc therapy and volumetric modulated arc therapy for treating single brain metastases: A retrospective analysis of dosimetric and clinical outcomes. Phys. Imaging Radiat. Oncol..

[40] Huq M.S., Hwang M.-S., Teo T.P., Jang S., Heron D.E., Lalonde R. (2018). A dosimetric evaluation of the IAEA-AAPM TRS483 code of practice for dosimetry of small static fields used in conventional linac beams and comparison with IAEA TRS-398, AAPM TG51, and TG51 Addendum protocols. Med. Phys..

[41] Behmadi M., Toossi M.T.B., Nasseri S., Ravari M.E., Momennezhad M., Gholamhosseinian H., Mohammadi M., Mdletshe S. (2024). Calculation of Organ Dose Distribution (in-field and Out-of-field) in Breast Cancer Radiotherapy on RANDO Phantom Using GEANT4 Application for Tomographic Emission (Gate) Monte Carlo Simulation. J. Med. Signals Sens..

[42] Bednarz B., Xu X.G. (2009). Monte Carlo modeling of a 6 and 18 MV Varian Clinac medical accelerator for in-field and out-of-field dose calculations: Development and validation. Phys. Med. Biol..

[43] Lawler G., Leech M. (2017). Dose Sparing Potential of Deep Inspiration Breath-hold Technique for Left Breast Cancer Radiotherapy Organs-at-risk. Anticancer Res..

[44] Das Majumdar S.K., Amritt A., Dhar S.S., Barik S., Beura S.S., Mishra T., Muduly D.K., Dash A., Parida D.K. (2022). A Dosimetric Study Comparing 3D-CRT vs. IMRT vs. VMAT in Left-Sided Breast Cancer Patients After Mastectomy at a Tertiary Care Centre in Eastern India. Cureus.

[45] Popescu C.C., Olivotto I.A., Beckham W.A., Ansbacher W., Zavgorodni S., Shaffer R., Wai E.S., Otto K. (2010). Volumetric modulated arc therapy improves dosimetry and reduces treatment time compared to conventional intensity-modulated radiotherapy for locoregional radiotherapy of left-sided breast cancer and internal mammary nodes. Int. J. Radiat. Oncol. Biol. Phys..

[46] Wesolowska P., Georg D., Lechner W., Kazantsev P., Bokulic T., Tedgren A.C., Adolfsson E., Campos A.M., Alves V.G.L., Suming L. (2019). Testing the methodology for a dosimetric end-to-end audit of IMRT/VMAT: Results of IAEA multicentre and national studies. Acta Oncol..

[47] McDonald A.M., Schneider C.S., Stahl J.M., Oster R.A., Popple R.A., Mayo C.S. (2023). A focused review of statistical practices for relating radiation dose-volume exposure and toxicity. Radiat. Oncol..

[48] Desideri I., Loi M., Francolini G., Becherini C., Livi L., Bonomo P. (2020). Application of Radiomics for the Prediction of Radiation-Induced Toxicity in the IMRT Era: Current State-of-the-Art. Front. Oncol..

[49] Bruand M., Salleron J., Guihard S., Crety C.M., Liem X., Pasquier D., Lamrani-Ghaouti A., Charra-Brunaud C., Peiffert D., Clavier J.-B. (2022). Acute skin toxicity of conventional fractionated versus hypofractionated radiotherapy in breast cancer patients receiving regional node irradiation: The real-life prospective multicenter HYPOBREAST cohort. BMC Cancer.

[50] Inada M., Nishimura Y., Ishikura S., Ishikawa K., Murakami N., Kodaira T., Ito Y., Tsuchiya K., Murakami Y., Saito J. (2022). Organs-at-risk dose constraints in head and neck intensity-modulated radiation therapy using a dataset from a multi-institutional clinical trial (JCOG1015A1). Radiat. Oncol..

[51] Bartlett G.K., Njeh C.F., Huang K.C., DesRosiers C., Guo G. (2023). VMAT partial arc technique decreases dose to organs at risk in whole pelvic radiotherapy for prostate cancer when compared to full arc VMAT and IMRT. Med. Dosim..

[52] Tai D.T., Oanh L.T., Son N.D., Loan T.T.H. (2018). Evaluation of jaws-only intensity modulated radiation therapy treatment plans using Octavius 4D system. Pol. J. Med. Phys. Eng..

[53] Guan F., Donahue W., Biggs S., Jennings M., Draeger E., Chen H., Wang Y., Nguyen N., Carlson D.J., Chen Z. (2024). 3D gamma analysis between treatment plans for nominally beam-matched medical linear accelerators using PyMedPhys. Precis. Radiat. Oncol..

[54] Chang Z., Wu Q., Adamson J., Ren L., Bowsher J., Yan H., Thomas A., Yin F.F. (2012). Commissioning and dosimetric characteristics of TrueBeam system: Composite data of three TrueBeam machines. Med. Phys..

[55] Stelljes T.S., Harmeyer A., Reuter J., Looe H.K., Chofor N., Harder D., Poppe B. (2015). Dosimetric characteristics of the novel 2D ioni-zation chamber array OCTAVIUS Detector 1500. Med. Phys..

