# Supplementary material for: Platform-Dependent Differences in Beam Characteristics and Low-Dose Exposure: A Comparative Study of Elekta™ Synergy and Varian TrueBeam™ Linear Accelerators Using SunSCAN™ 3D Phantom and Octavius® 4D QA
Source: J Clin Med. 2026 Feb 20;15(4):1619. doi: 10.3390/jcm15041619 (PMC12942439; doi:10.3390/jcm15041619)
Supplement: Supplementary file 1 [file jcm-15-01619-s001.zip › jcm-4089655-supplementary.pdf]

Journal of Clinical Medicine

MDPI

Supplementary Materials

Table S1. Homogeneity Index (HI) – TrueBeam vs Elekta (comparative).

Clinical site	HI (TrueBeam)	HI (Elekta)	ΔHI (TB – Elekta)
Breast (Left)	0.095	0.109	-0.014
Prostate	0.046	0.068	-0.022
H&N	0.069	0.086	-0.017

Table S2. Planned OAR dose–volume metrics for left-sided breast IMRT plans.

Structure	Metric	Planning goal*	TrueBeam	Elekta	Δ (TB–Elekta)	Units
Heart	Dmean	< 4–5	6.07	1.07	5	Gy
Heart	V25Gy	< 10	4.8	3.12	1.68	%
Ipsilateral lung	V20Gy	< 35	26.6	32.84	−6.24	%
Ipsilateral lung	V5Gy	< 65	81	77	4	%
Contralateral lung	V5Gy	< 15	15	17	−2.0	%
Contralateral breast	Dmean	< 5	3.3	3.5	−0.20	Gy
Spinal cord	Dmax	< 30	25.49	15	10.49	Gy

Table S3. Planned OAR dose–volume metrics for prostate plans.

Structure	Metric	Planning goal*	TrueBeam	Elekta	Δ (TB–Elekta)	Units
Femoral head (Right)	Dmax	< 52	48.1	48.8	−0.70	Gy
Femoral head (Left)	Dmax	< 52	48.35	47.9	0.45	Gy
Rectum	V70Gy	< 10-15–20	9.8	17	−7.2	%
Rectum	V60Gy	< 35	22.8	42.44	−19.64	%
Rectum	V50Gy	< 50	47	46.39	0.61	%
Bladder (with overlap)	V70Gy	< 25	21	25	−4.0	%
Bladder (with overlap)	V60Gy	< 35–40	34	42.44	−8.44	%
Bladder (with overlap)	V50Gy	< 50	52	77.69	−25.69	%
Bladder (excluded overlap)	V70Gy	< 15	3	20	−17.0	%
Bladder (excluded overlap)	V60Gy	< 25	17	41	−24.0	%
Bladder (excluded overlap)	V50Gy	< 30	41.1	55	−13.9	%
Bowel / Intestine	V45Gy	< 195 CC	159.9	163	-3.1cc	

Table S4. H&N (Oropharynx) – plan sum (54 Gy + 6 Gy + 6 Gy): comparative OAR dose metrics and planning goals.

Structure	Metric	Planning goal*	TrueBeam	Elekta	Δ (TB–Elekta)	Units
Spinal cord	Dmax	< 45	38.56	39.52	−0.96	Gy
Spinal cord PRV	Dmax	< 50	44.95	42.7	2.25	Gy
Parotid (Right)	Dmean	< 26	22.97	20.92	2.05	Gy
Parotid (Left)	Dmean	< 26	22	24.72	−2.72	Gy
Esophagus	V35Gy	As low as reasonably achievable	37.815	31.96	5.855	%
Mandible	Dmax	< 70	43	40.4	2.6	Gy
